# Internet addiction induced critical fusion frequency among young adults

**DOI:** 10.6026/97320630019816

**Published:** 2023-07-31

**Authors:** Jagadamba Aswathappa, MV Shobha, Pendyala Manogna, Lingamaneni Himavarshini

**Affiliations:** Sri Devrajurs Academy of Higher Education & Research, Tamaka, kolar-563101, Karnataka, India

**Keywords:** critical fusion frequency, internet addiction

## Abstract

Critical flicker fusion frequency (CFF) is the frequency at which flickering light can be perceived as continuous and it is used to assess the processing of temporal vision. It is of interest to compare critical fusion frequency (CFF) in mild, moderate & severe internet users. Cross sectional observation study was done among 350 professional students. Internet addictions for around 372 professional students & internet addiction was evaluated by Young's scale of Internet Addiction questionnaire in the age group 18-25 years. CFF was measured using an in-house built LED-based CFF M1 Model instrument. A total of 372 participants (in age group 19-22 years) were recruited. Of the participants 65 % of them were mild internet addictors, 48% were moderate and 7% were severe internet adductors. Among the internet usage, the Kruskal-Wallis test showed a significant difference in internet addiction score, duration of internet usage (hours) and average Critical fusion frequency (Hz) (P<0.001). For the average critical fusion frequency (Hz), the Dwass-Steel-Critchlow-Fligner test showed significant pairwise comparisons between the following: (1) mild internet addictor's vs moderate internet addictors and (2) Mild internet addictors versus severe internet addictors (p<0.001). Critical flicker fusion frequency(CFF) as it is an easy, quick, and non-invasive technique used as a general indicator of cortical processing, visual persistence for cognitive flexibility.

## Background:

Internet usage among the general population is increasing widely. According to Internet World stats, up to November 2015, there were around 3.36 billion users. [1] Among Internet users, young adults are widely using the Internet & getting addicted to the same. Young defined internet dependence as an impulse control disorder that does not involve intoxicants, and an individual loses control of internet use to cause significant relational, occupational, and social impairments [3]. Mental health can also be affected by engaging in the usage of the Internet at an early age. Adolescence is a critical period of brain development, and excessive internet use can have a significant impact on this process. This period is marked by heightened vulnerability to various mental health issues, including affective disorders and addiction. [8] The pathogenesis of Internet addiction involves complicated and multiple psychopathological and behavioural symptoms. [5] As with heavy Internet usage, asked the respondents whether they experienced restlessness, irritability, anxiety, and low mood when staying on the Internet for a long time. [6] Internet addiction on cognitive performance by radio imaging has shown less efficient information processing & abnormal spontaneous brain activity with poor performance. [7] The CFF is widely used in studying human behaviour physiology of vision. CFF (Critical fusion frequency) is an objective test applied to study the effect of addiction. It provides a measure to distinguish discrete sensory events and also provides an index of the central nervous system (CNS) activity or "cortical arousal.Studies have concentrated on subjective methods to assess internet addiction. Therefore, it is of interest to study the effect of internet addiction with CFF.

## Materials and Methods:

## Study Design:

The study was conducted using a cross-sectional observational design to collect data from professional students in the age group of 18-25 years. Written informed consent was obtained from all participants.

## Sample Size:

The sample size was estimated based on a prevalence of internet addiction of 35%. The formula used to calculate the sample size is:

n = [DEFF *Np (1-p)] / [(d^2 / Z^2) * (N-1) + p * (1-p)]

Using a 10% error margin and a 95% confidence interval, the calculated sample size is n = 372

## Interventions:

No specific interventions were implemented in this study. It aims to observe and collect data on internet addiction and its relationship with the Critical Fusion Frequency (CFF).After obtaining institutional ethical clearance and written informed consent from the participants, the data for the study was collected using a self-administered questionnaire. The questionnaire included the use of Young's scale of Internet Addiction, which is a reliable scale developed by Dr. Kimberly Young in 1998. [3]Young's scale of Internet Addiction is a commonly used tool to assess the level of internet addiction in individuals. It consists of a series of questions that measure various aspects of internet use and its impact on daily life. Participants are asked to rate their responses based on a predefined scale, which allows for the quantification of internet addiction levels. Internet addiction for around 350 professional students was evaluated by Young's scale of Internet Addiction. Then they were classified into mild (20-49 points); moderate (50-79) & severe internet addictors (80-100) based on the score obtained by the questionnaire.

## Measurement of CFF:

The CFF was measured using an in-house built LED-based CFF M1 Model instrument. The instrument consists of two components. The first component is a flickering light source placed on a white background, providing central field stimulation. The light source will be presented separately to each eye by covering the other eye. A monochromatic red light with a wavelength of 630nm will be used, as it is perceived for a longer time in the retina. The on and off periods of the light source are equal, eliminating any delay period for switching on. The second component is a variable frequency square wave oscillator that can generate oscillations in the range of 10-80 hertz with an accuracy of 0.5 hertz. To measure the CFF, the examination room was partially illuminated, and the subject was seated comfortably. They will be presented with the red light source at a distance of 25-30cms. The frequency of oscillations was gradually increased, and the subject was instructed to respond when the flickering light source appears as a single fused light. The frequency at which this occurs is recorded as the critical flicker fusion frequency (CFF). The frequency was measured from the recorded data using Sweepgen software. The CFF was measured separately for the right and left eye, and the average of the two frequencies was considered as the final CFF value for each subject. Individuals with a history of blindness, diabetes mellitus, hypertension, or cataracts were excluded from the study. [9]

## Statistical analysis:

In the quantitative data analysis, descriptive statistics were computed as Mean +SD to summarize the data and provide an overview of the variables of interest. To assess the normality of the continuous response variables, Shapiro-Wilk test was conducted. As the data did not satisfy the normality requirements as indicated by the Shapiro-Wilk test, non-parametric tests was employed to compare the Critical Fusion Frequency (CFF) among different groups, such as internet adductors by Kruskal-Wallis test. It tests the null hypothesis that the population medians of the compared groups are equal. Following the Kruskal-Wallis test, the Dwass-Steel-Critchlow-Fligner (DSCF) test was conducted for all possible pair comparisons between different groups. The DSCF test is a non-parametric multiple comparison test that determines statistically significant differences between pairs of groups. This post-hoc test helps identify which specific groups differ significantly from each other. The statistical software Jamovi for Windows version 2.3.26 was used to perform these analyses. The significance level for determining statistical significance was set at p < 0.05.

## Results:

Based on the Shapiro-Wilk tests, all the variables mentioned in Table 1 exhibit significant departures from normality internet addiction score (W = 0.979, p < 0.001), Right eye critical fusion frequency (W = 0.943, p < 0.001), and left eye critical fusion frequency (W = 0.979, p < 0.001),age (W = 0.923, p < 0.001) & average critical fusion frequency(W = 0.970, p < 0.001), duration of internet usage (W = 0.966, p < 0.001),according to Shapiro-Wilk tests (Table 1). A total of 372 participants (in age group 19-22 years) were recruited. Of the participants 65 % of them were mild internet addictors, 48% were moderate, 7% were severe internet adductors. Duration of internet usage among severe internet addictors is more 12.92+1.74 hours & Average critical fusion frequency (Hz) is 49.5+5.48 Hz in severe internet addictors (Table 2). Among the internet usage, the Kruskal-Wallis test showed a significant difference in Internet addiction score, Duration of internet usage (hours) , Average Critical fusion frequency(Hz) (P<0.001) (Table 3). Table 4 shows Dwass-Steel-Critchlow-Fligner (DSCF), test for multiple comparisons analysis of the differences between the median values of the internet addiction in the three groups. For the duration of internet usage, the Dwass-Steel-Critchlow-Fligner test showed significant pairwise comparisons between the following: mild internet addictor’s vs severe internet addictors and moderate internet addictors VS severe internet addictors (p<0.001) (Table 4). For the internet addiction score, the Dwass-Steel-Critchlow-Fligner test showed significant pairwise comparisons between the following: mild internet addictor's vs severe internet addictors and moderate internet addictors VS severe internet adductors & mild internet adductors & severe internet addictors (p<0.001) (Table 5). For the average critical fusion frequency (Hz), the Dwass-Steel-Critchlow-Fligner test showed significant pairwise comparisons between the following: mild internet addictor's vs moderate internet addictors and Mild internet addictors VS severe internet addictors (p<0.001) (Table 6).

## Discussion:

In the present study, there was a significant increase in the internet addiction score among severe internet addicts, comprising around 7%. Internet addiction, also known as pathological use of the Internet, is characterized as an impulse-control disorder that negatively affects a person's functioning across various areas of their life. Individuals with PIU may experience impairments in their ability to control their attention, inhibit certain motor responses, make effective decisions, and retain information in working memory. [10] The findings suggest that prolonged exposure to heavy smartphone use can negatively impact arithmetic accuracy. This implies that excessive smartphone usage can interfere with cognitive processes related to mathematical calculations and problem-solving. [11] In the present study duration of internet usage among severe internet addicts is significantly higher. There was a significant decrease in average CFF in the severely internet addict group, which can be well correlated with significance. Individuals with moderate addiction to the Internet, combined with impaired cognitive flexibility, may face challenges in controlling their internet use. When cognitive flexibility is impaired, individuals may struggle to modify their internet usage patterns despite recognizing the negative consequences of their addiction. [12] This decreased sensitivity can lead to reduced plasticity in the visual areas of the brain, impairing the brain's ability to adjust & change in response to visual stimuli. These changes in CFF and visual processing can contribute to cognitive fatigue, which refers to a state of mental exhaustion or weariness. Cognitive fatigue can manifest as a failure to sustain attention and optimize task performance during prolonged mental effort. Internet addiction linked with structural and functional changes in specific brain areas. These changes involve both cortical regions, such as the prefrontal cortex (PFC) and limbic structures, as well as subcortical regions, including parts of the basal ganglia. The alterations observed in these cortical and limbic regions suggest that internet addiction may impact cognitive and emotional processes. The alterations in brain regions involved in executive functions, such as the prefrontal cortex (PFC), suggest that internet addiction may lead to deficits in these cognitive processes. [13] Given these findings, intervention strategies targeting executive function processes, such as cognitive flexibility, could be valuable in addressing internet addiction. By enhancing cognitive flexibility, individuals become better equipped to adapt their behaviour, resist impulses, and make healthier choices regarding their internet use.

## Conclusion:

In conclusion of this study, critical flicker fusion frequency(CFF) as it is an easy, quick, and Non-invasive technique used as a general indicator of cortical processing, visual persistence, and perceptual learning on Internet addiction is an increasing problem among professional students, which has psychological, physical & social impact on students life. It is necessary to develop awareness, prevention strategies, and therapeutic interventions among professional students, which is essential for promoting healthy & safe use of the Internet.

## Figures and Tables

**Table 1:** Normality Test (Shapiro-Wilk)

**Variables**	**W**	**p**
Age(years)	0.923	<.001
Internet addiction score	0.979	<.001
Duration of internet usage/day(Hours)	0.966	<.001
Right eye critical fusion frequency(Hz)	0.943	<.001
Left eye critical fusion frequency(Hz)	0.979	<.001
Average critical fusion frequency	0.97	<.001

**Table 2:** Descriptive statistics among the 3 groups of internet addictors

**Variables**	**Mild internet addiction n=242 (65%) (Mean±SD)**	**Moderate internet addiction n=106(48%) (Mean±SD)**	**Severe internet addiction n=24(7%) (Mean±SD)**
Age (Years)	19.80±1.91	20.02±1.97	22.25±1.45
Internet addiction score	32.06±9.94	56.39±6.84	86.67±5.16
Duration of internet usage (hours)	6.35±2.34	6.65±2.74	12.92±1.74
Right eye Critical fusion frequency(Hz)	27.54±2.87	25.27±3.03	24.71±2.73
Left eye Critical fusion frequency(Hz)	27.2±2.59	25.49±3.19	24.79±2.86
Average Critical fusion frequency(Hz)	54.74±5.14	50.76±5.97	49.5±5.48

**Table 3:** Differences between the median values of the internet addictors in the three groups were analyzed by the Kruskal-Wallis non-parametric test

	**χ²**	**df**	**p**	**ε²**
Internet addiction score	260.6	2	<.001	0.7024
Duration of internet usage (hours)	63.5	2	<.001	0.1712
Right eye Critical fusion frequency(Hz)	50.2	2	<.001	0.1352
Left eye Critical fusion frequency(Hz)	41.6	2	<.001	0.1121
Average Critical fusion frequency(Hz)	48.2	2	<.001	0.1299

**Table 4:** Pairwise comparisons - Duration of internet usage (hours)

		**W**	**P**
mild internet addictors	moderate internet addictors	0.701	0.874
mild internet addictors	Severe internet addictors	11.251	<.001
moderate internet addictors	Severe internet addictors	10.118	<.001
W = Wilcoxon rank sum test statistic

**Table 5:** Pairwise comparisons - internet addiction score

		**W**	**P**
mild internet addictors	moderate internet addictors	21	<.001
mild internet addictors	Severe internet addictors	11.4	<.001
moderate internet addictors	Severe internet addictors	10.9	<.001

**Table 6:** Pairwise comparisons - Average Critical fusion frequency (Hz)

		**W**	**P**
mild internet addictors	moderate internet addictors	-8.78	<.001
mild internet addictors	Severe internet addictors	-5.72	<.001
moderate internet addictors	Severe internet addictors	-1.7	0.451
